# IgA nephropathy and the road to motherhood

**DOI:** 10.1093/ckj/sfag146

**Published:** 2026-05-09

**Authors:** Eleni Stamellou, Anila Duni, Evangelia Dounousi

**Affiliations:** Department of Nephrology, School of Medicine, University of Ioannina, Ioannina, Greece; Division of Nephrology and Rheumatology, RWTH Aachen University Hospital, Aachen, Germany; Department of Nephrology, School of Medicine, University of Ioannina, Ioannina, Greece; Department of Nephrology, School of Medicine, University of Ioannina, Ioannina, Greece

To the Editor,

IgA nephropathy (IgAN) is the most common primary glomerulonephritis worldwide, with its incidence peaking during a woman’s childbearing years [1]. Despite this epidemiological reality, the reproductive implications of IgAN remain profoundly underappreciated in routine clinical practice. We report a case that illustrates a principle we believe warrants broader recognition: every diagnosis of IgAN in a young woman must immediately prompt a structured discussion about future pregnancy risks and reproductive planning—not as a secondary consideration, but as a core component of the initial clinical encounter and the long-term holistic approach.

A woman in her early thirties was found to have microhematuria on routine screening. No nephrology workup was initiated at that time. At 38, having completed postgraduate studies in the intervening years, she became pregnant. Her pregnancy was complicated by severe preeclampsia and Hemolysis, Elevated Liver enzymes and Low Platelets (HELLP) syndrome at gestational week 16, necessitating the early termination with fetal loss. Four weeks after pregnancy termination, serum creatinine was 1.5 mg/dl and urinary total protein excretion 3.5 g/day. Subsequent renal biopsy confirmed IgAN, with 42% global glomerulosclerosis and Oxford score M1E1S1T1. Following diagnosis, corticosteroid therapy yielded an initial response, but proteinuria relapsed within 1 month of treatment cessation. A subsequent course of targeted-release budesonide provided no meaningful benefit. Finally, with maximal supportive therapy, estimated glomerular filtration rate (CKD-EPI 2021) stabilized at 39 ml/min/1.73 m^2^. Multidisciplinary assessment concluded that further pregnancy posed very high risk to both maternal health and residual kidney function. She therefore pursued gestational surrogacy as a medically indicated pathway rather than an elective choice. She underwent multiple cycles of ovarian stimulation for oocyte retrieval, each associated with uncertainty regarding the effects of exogenous hormones on blood pressure and proteinuria.

Beyond the medical challenges, she encountered a formidable legal and administrative landscape. Engaging legal counsel, navigating court proceedings, obtaining the required certifications, and identifying a suitable and accredited surrogacy center demanded months of effort, emotional resilience, and significant financial resources.

This case compels us to reflect on several systemic failures. First, the absence of early nephrology referral for a young woman with microhematuria represents a missed opportunity for early diagnosis, targeted therapy, and—most critically—timely preconception counseling. Women with IgAN carry a more than four-fold increased risk of preeclampsia compared to the general population [2–4], while pregnancy in women with chronic kidney disease (CKD) stages 3–5 can shorten time to renal replacement therapy by 2.5–4.7 years [5].

Second, the lack of evidence-based guidance on the safety of ovarian stimulation in women with stage 3 CKD and active proteinuria leaves both clinicians and patients navigating uncharted territory; each stimulation cycle becomes an undocumented experience, with no systematic data collection and no means of protecting the next patient.

Third, the financial, legal, and emotional burden required to pursue surrogacy reveals a profound inequity: access to biological parenthood for women with kidney disease is determined not only by their medical profile, but by their socioeconomic circumstances. In East-European countries, the costs of surrogacy—which can reach or exceed €70 000—fall entirely outside the scope of public health coverage, making this path to parenthood unattainable for many.

Although the most recent Kidney Disease: Improving Global Outcomes guidelines address pregnancy in CKD for the first time, available data remain limited [6, 7]. We contend that the burden of IgAN in young women extends well beyond disease treatment—it encompasses reproductive futures, paths to parenthood, and life trajectories that current nephrology care is not yet structured to support. When IgAN is diagnosed in a young woman of reproductive age, pregnancy risk stratification, fertility counseling, and conception timing relative to disease activity and treatment must be integrated from the moment of diagnosis. Dedicated clinical pathways as published for patients with systemic lupus erythematosus [8], prospective data collection on assisted reproduction in CKD, and formal multidisciplinary collaboration between nephrology and reproductive medicine represent urgent and unmet needs for this patient population.
